# Preservation of Flowers

**Published:** 1861-02

**Authors:** 


### Preservation of Flowers.

It is stated in a French paper, that flowers may be kept fresh for any length of time, by introducing into the vessel of water which contains them, a spoonful of pulverized charcoal.

